# Neuropathic foot ulcers in the tallest patients with acromegalic gigantism: a common and significant problem. Historical overview

**DOI:** 10.1007/s11102-023-01325-4

**Published:** 2023-06-04

**Authors:** Wouter W. de Herder, Warren A. Raymond

**Affiliations:** 1 Department of Internal Medicine, Sector of Endocrinology, Erasmus MC, Dr. Molewaterplein 40, 3015 GD Rotterdam, The Netherlands; 2 Silver Spring, Cloverly, MD 20905 USA

**Keywords:** Acromegaly, Gigantism, Neuropathy, Foot ulcer, History

## Abstract

**Purpose:**

We present a historical overview on neuropathic ulcers in patients with acromegalic gigantism.

**Materials and methods:**

The case histories of 6 famous patients with acromegalic gigantism and living in the twentieth century were analyzed. The combined final height and maximum weight of these giants were: 272 cm. & 215.9 kg., 218.4 cm. & 125 kg., 242 cm. & 165 kg., 220.5 cm. & 135 kg., 235 cm. & 136 kg. and 224.8 cm. & 174 kg.

**Conclusions:**

Neuropathic foot ulcers leading to hospital admissions and surgical and medical interventions were reported in 6 patients with acromegalic gigantism. These ulcers significantly impaired the daily activities of these individuals. Neuropathies of the sural nerve in patients with acromegalic gigantism can lead to hypoesthesia and hypoalgesia of the lower legs and feet. Potential contributing factors for the development of neuropathic ulcers of the feet in patients with acromegalic gigantism and neuropathy might be leg and foot deformities, muscle weakness and poor quality footwear. Diabetes mellitus, or impaired glucose intolerance does not necessarily seem to play a role.

## Robert Wadlow, the tallest man ever

**Fig. 1:**
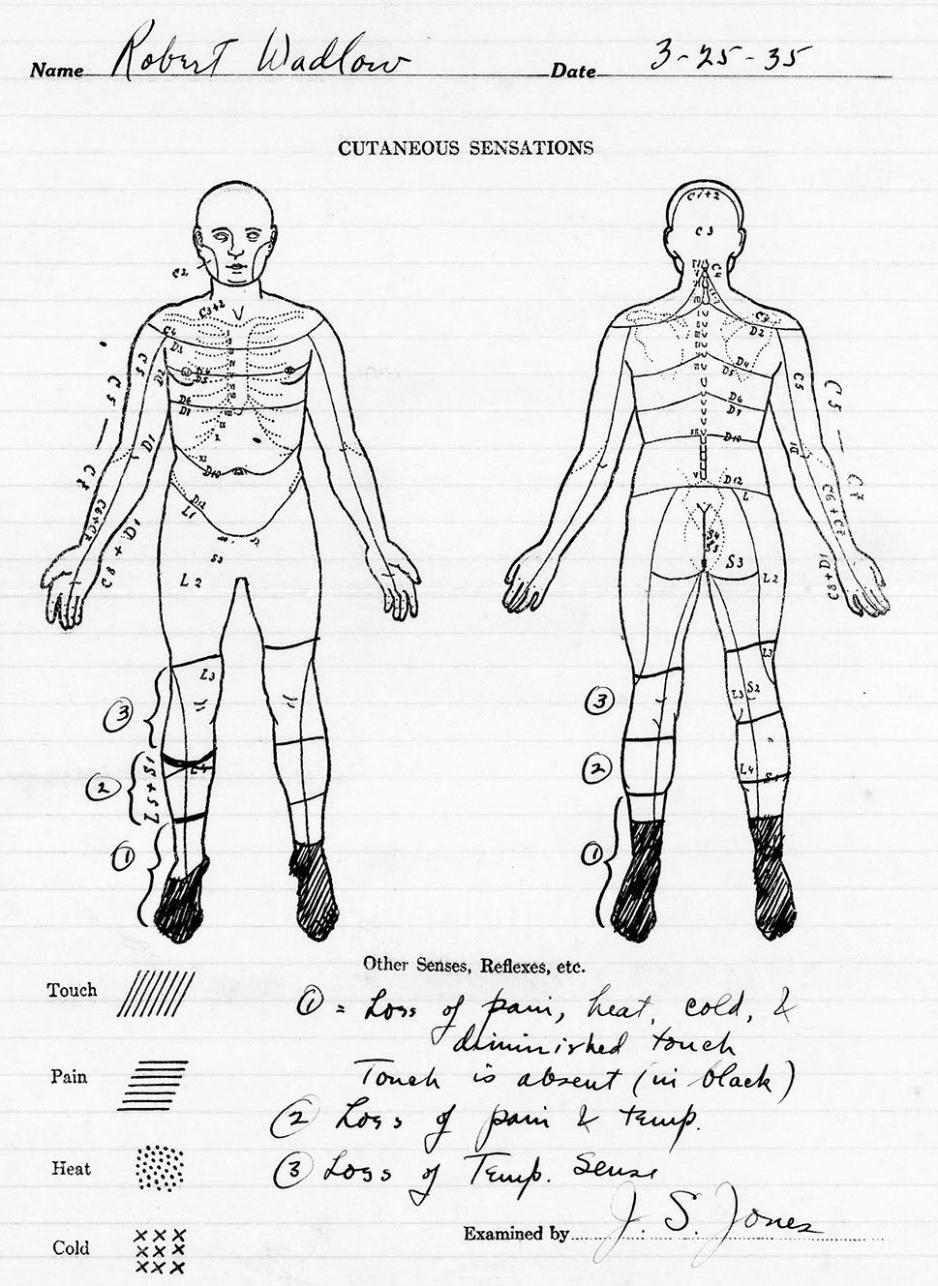
Schematic recording of the “cutaneous sensations” of the American acromegalic giant Robert Wadlow (1918–1940) dating 25 March 1935—age 17 years & 1 month (height 244 cm., weight 170 kg.)[12]—demonstrating sensory defects in the lower legs. Picture from the Barnes hospital, St. Louis, MO hospital medical file—private collection Warren A. Raymond

**Fig. 2:**
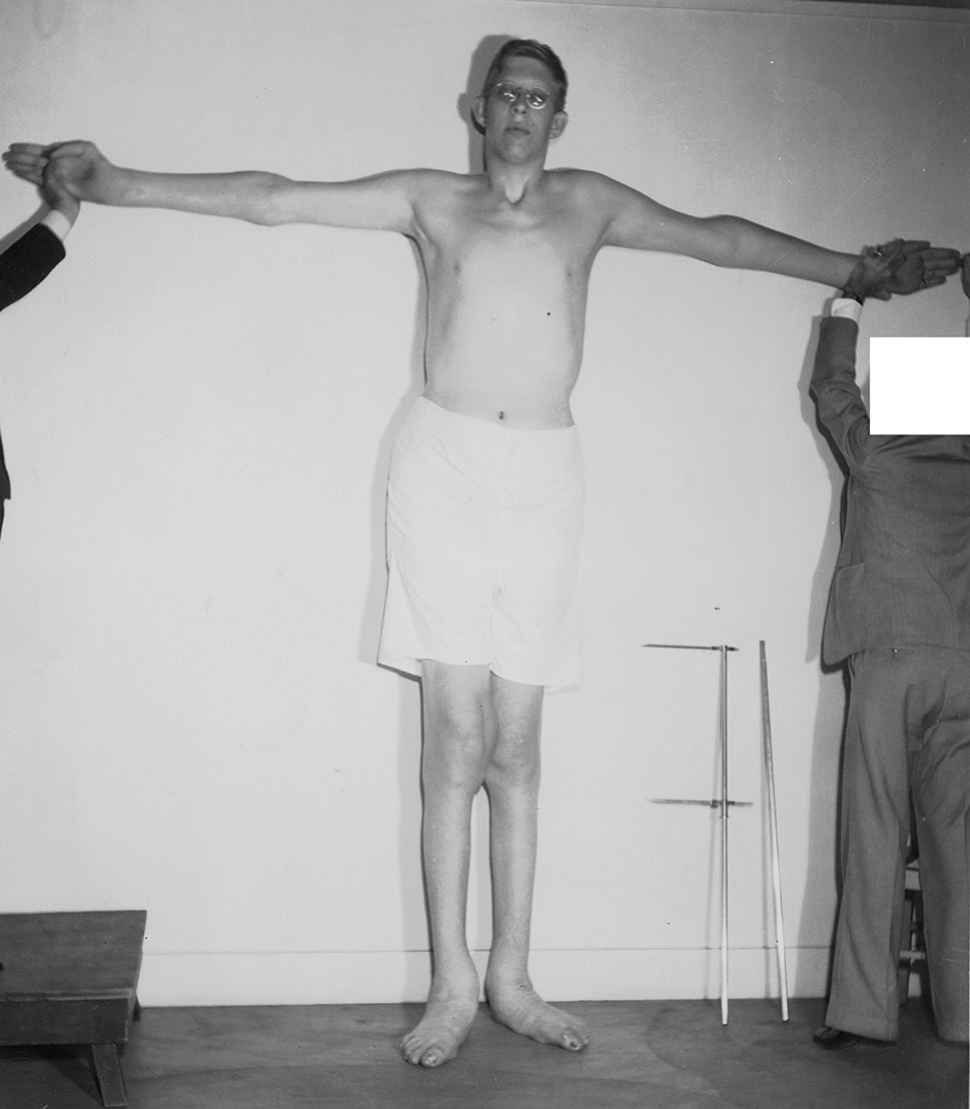
Photograph of the American acromegalic giant Robert Wadlow (1918–1940) dating 19 October 1936—age 18 years & 8 months (height 255.9 cm., weight 197.7 kg.)[12]—showing deformities of both legs (genu valga), knees and feet. Photograph from the private collection of Warren A. Raymond. The bottom part of this photograph, only showing the legs, was also reproduced in the paper by Daughaday, et al. [12]

In their paper “Hyperpituitarism beginning in infancy, the Alton giant”, published in 1932, the physicians Louis Henry Behrens (1868–1948) and David Preswick Barr (1889–1977) of the St. Louis Barnes Hospital report on a remarkable case of acromegalic gigantism which started to develop between ages 1 and 2 years old [1]. The patient, *Robert Pershing Wadlow*, was born on February 22, 1918 in Alton, IL, USA and at the time of the case report he had been followed up between the ages 11 years & 11 months and 13 years & 6 months old. During this short period his height increased from 208 to 221.5 cm. [1]. The publication does not mention sensory deficits, nor ulcers, or healing problems of the lower extremities in this young man with gigantism. Robert Wadlow would continue to grow to finally become the tallest man ever with a final height of 272 cm. (8′ 11.1″) [2–9]. His maximum weight was 215.9 kg. He died on 15 July 1940 at the age of 22 years & 5 months. His untimely death occurred due to an infected abrasion on his ankle caused by a leg brace that was required because of peroneal nerve paralysis (drop foot) leading to cellulitis and bacteremia. In that era the first antibiotic, penicillin, was not yet readily available; indeed the first British patient was treated with penicillin on February 12, 1941, after which it was also introduced in the United States [10]. Being the private patient of the surgeon Franklin E Walton (1902–1981), Robert Wadlow was admitted several times at Barnes Hospital in St Louis, MO, USA for foot infections. The hospital records mention four hospital admissions starting in 1931. From 13 to 23 October 1931, he was hospitalized because of cellulitis of the left foot, following an injury, for which he underwent incision and drainage. He was readmitted from 21 June to 2 July 1932 because of an infection of the big toe of the left foot and again in 1932 from 18 to 23 October for another infection of the same toe and a fracture of the left 2nd metatarsal bone after an injury. Finally, an abscess was incised and drained. The hospital files report on another hospital admission from 24 March 1935 to 4 May 1935 because of an ulcer of the big toe of the right foot and cellulitis. At that time he was 17 years old and his height at admission was 244 cm. He weighed 149 kg., although his previously reported weight was 170 kg. It was suggested that because of a diminished food intake/loss of appetite he had lost weight before and during hospital admissions. At a certain stage the diagnosis of “hypophysial cachexia, Simmonds’ disease” was considered. Robert Wadlow was only treated with desiccated thyroid hormone. Hydrocortisone/cortisol was not available at the time; the US chemist Edward Calvin Kendall (1886–1972) was isolating compound E (later renamed cortisone), which was subsequently synthetized in 1946 [11]. In 1948, the first patient with rheumatoid arthritis was treated with cortisone by the US rheumatologist Philip Showalter Hench (1896–1965) [11]. During hospitalization, Robert Wadlow was also under the continuous care of Dr Barr. Already in October 1932, it was reported that reflexes in the lower extremity were “difficult to obtain”. In 1935, vibratory, temperature and pain sensation in the feet, ankles and lower legs were reduced and reflexes were absent (Fig. 1). Because of multiple joint problems in the legs and pelvis in combination with muscle weakness, his leg movements became progressively impaired. In the last years of his life, Robert Wadlow had to use one or two canes for assistance in walking. In pictures and short movies, genu valga (knock knees) are apparent with swollen knee and ankle joints and markedly deformed feet (Fig. 2). It should be noted that diabetes mellitus, or impaired glucose tolerance was absent. In 1977, the Endocrinologist William Hamilton Daughaday (1918–2013), from the Department of Medicine, Washington University School of Medicine at St. Louis, MO, retrospectively reported on these findings [12]. Daughaday also suggested that, based on the hospital records, Robert Wadlow developed carpal tunnel syndrome leading to difficulties holding a pen or pencil [12].

## Other famous tall individuals with acromegalic gigantism in the twentieth century

**Fig. 3:**
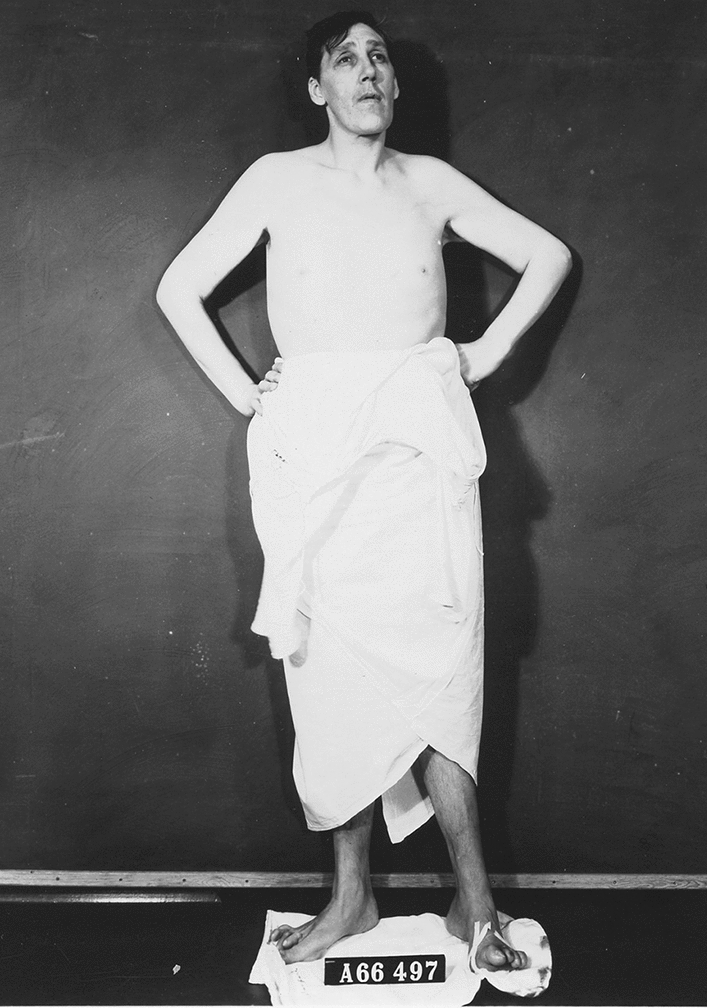
Photograph of the American acromegalic giant Johann (John) Aasen (1890–1938—final height of 218.4 cm.) being hospitalized in Lane Hospital in San Francisco (CA) because of recurrent bilateral foot ulcers—2 November 1936 [13]. Recorded height at admission was 213.4 cm. [13]. Note the foot deformities (clawing toes). A penetrating ulcer on the bottom of the left foot is not shown. Photograph from the private collection of Warren A. Raymond. This photograph was also partly reproduced in a different manner in the paper by Gray, et al. [13]

The US acromegalic giant artist/movie actor *Johann (John) Aasen* (March 5, 1890–August 1, 1938, the “Minneapolis giant”, the “Harold Lloyd giant”) finally reached a height of 218.4 cm. and weighted 125 kg. [3, 9, 13]. In 1936, at the age of 46 years, he was admitted to Lane Hospital in San Francisco (CA) because of recurrent bilateral foot ulcers of four years’ duration [13]. At physical examination he presented with clawing toes and a penetrating ulcer on the bottom of his left foot, without signs of osteomyelitis (Fig. 3). There was “marked hypesthesia and hypalgesia” over the ankles and the feet. The oral glucose tolerance test (OGTT) was normal for blood glucose measurements [13].

**Fig. 4:**
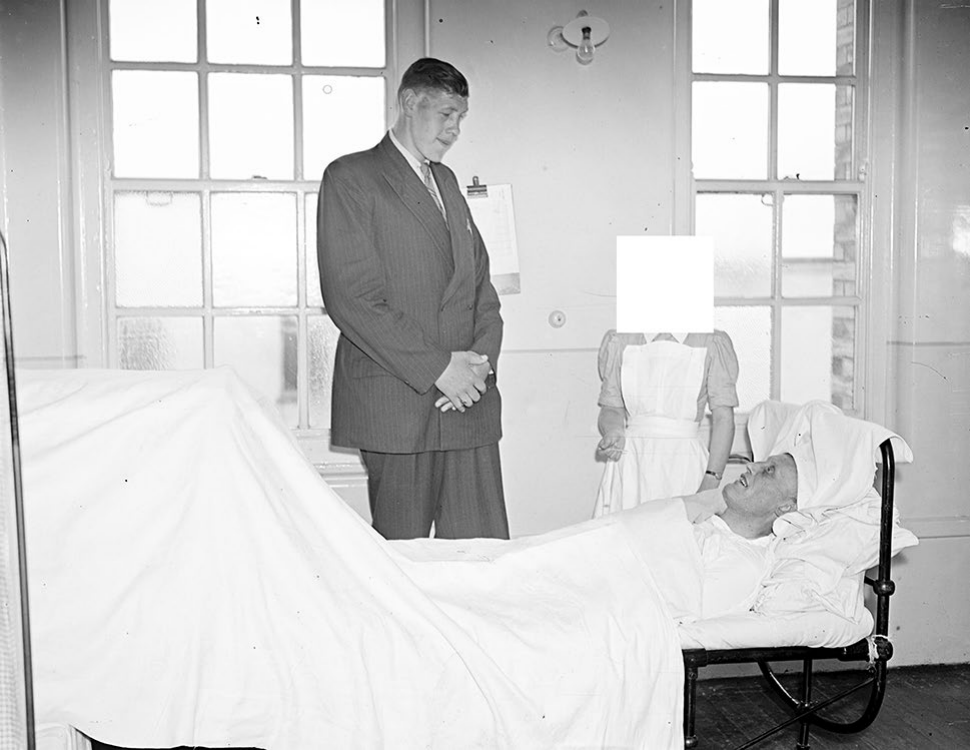
Photograph of the Dutch acromegalic giant Albert Johan Kramer (1897–1976, “Lofty”—final height 242 cm.) being hospitalized in Plymouth City Hospital, Plymouth, UK, because of “leg ulcers”—12 August 1948. Star of the show “Would You Believe it” (organizer Pete Collins (1908–1980)) at the Palace Theatre in Plymouth, he was being replaced by another Dutch acromegalic giant, Rigardus Rijnhout (1922–1959, final height 237.5 cm.) [3, 57] who was visiting him in the hospital. Photograph from the private collection of Wouter W. de Herder, which also appeared in the newspaper “De Leidse Courant” on 14 August 1948 (among other newspapers)

The tallest Dutchman and vaudeville artist *Albert Johan Kramer* (15 June 1897–4 April 1976, “Lofty”, “Jan van Albert”) was an acromegalic giant who was already 2 m. at the age of 7 years. His final height was 242 cm. and he weighed 165 kg. [3, 9, 14, 15]. Already at the age of 15 years, he developed a foot ulcer because of an injury. In the following years he had recurrent foot ulcers which sometimes required hospitalization [16, 17] (Fig. 4). Reports describe genu valga and bilateral hammer toes—one toe on the left foot had to be removed. Achilles tendon reflexes were difficult to elicit and there was a loss of muscle strength. He was not diagnosed with diabetes mellitus, or impaired glucose intolerance [3, 14, 15]. In later life he had to walk assisted by a cane. Eventually the amputation of both legs below the knees had to be performed [3].

**Fig. 5:**
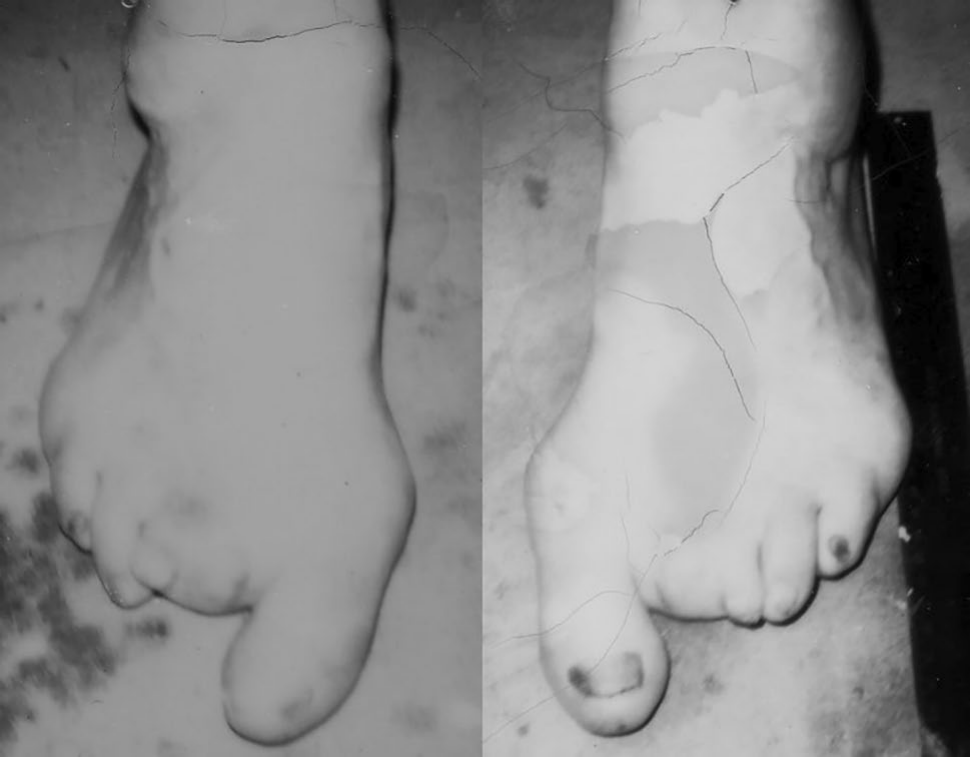
Photographs of the left and right foot of the Icelandic acromegalic giant Johann Petursson** (**9 February 1913–26 November 1984—final height 220.5 cm.) showing deformities and the situation after amputations of the 3rd and 4th toes, because of recurrent ulcerations and infections. Photographs from the private collection of Warren A. Raymond

The Icelandic sideshow performer and acromegalic giant *Johann Petursson* **(**9 February 1913–26 November 1984, Jóhann Svarfdælingur, “The Viking Giant”, “Jóhann Risi”, “Olaf”, “der Nordische Riese Olaf”) achieved a final height of 220.5 cm. and his maximum weight was 135 kg. [3, 9, 18–21]. Like so many other patients with acromegalic gigantism he also had back and joint problems. Already at the age of 20 years, he presented with foot ulcers and loss of sensation in the lower extremities was reported [18] (Fig. 5).

**Fig. 6:**
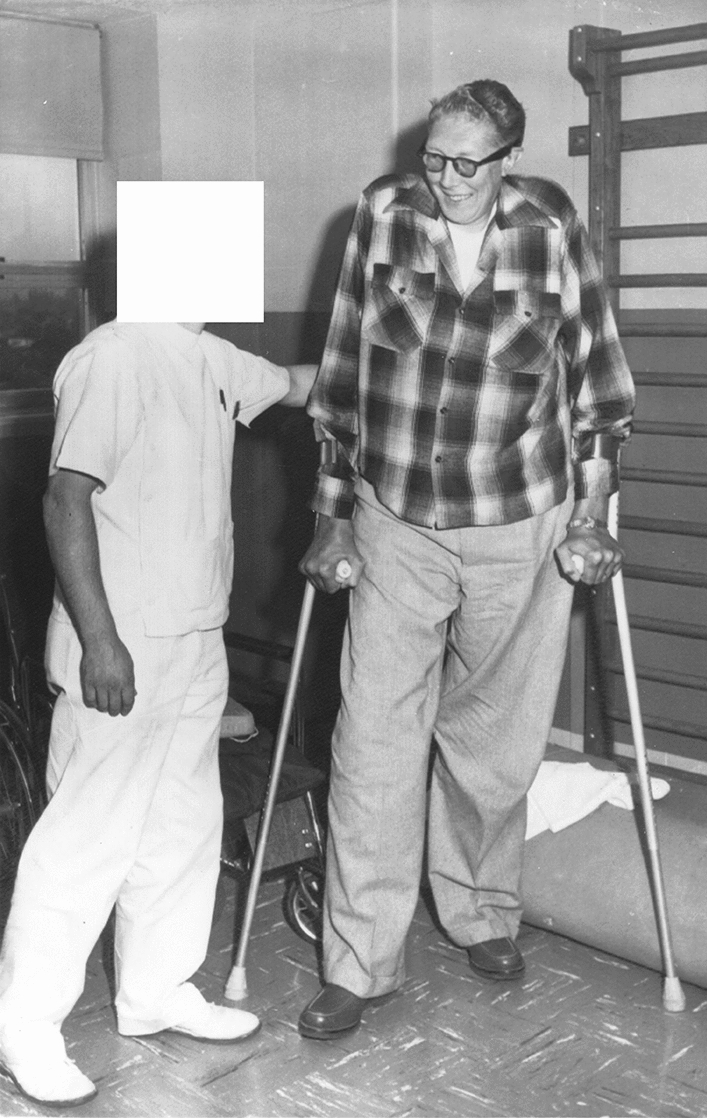
Photograph of the American acromegalic giant Cecil Boling (1920–2000—final height 235 cm.) learning to walk again on artificial legs (which reduced his height to 213 cm.) in The University Hospital in Seattle, WA, USA—2 July 1960. Photograph from the private collection of Wouter W. de Herder, which also appeared in the newspaper “The Joplin Globe” on 3 July 1960 (among other newspapers) [23]

The US acromegalic giant *Cecil Boling* (October 11, 1920–February 9, 2000, “Primo Boling”) started growing abnormally after the age of six years reaching a final height of 235 cm. [22]. He weighed 136 kg. Cecil Boling worked as a “tallest man” in the circus and as a musician he had his own band. He developed progressive varus deformities of both feet and already at the age of 18 years he was diagnosed with foot ulcerations. By the age of 21 years, he required leg braces in order to walk. Frequent hospitalizations for infections and osteomyelitis followed and eventually at the age of 28 years, he became wheelchair-bound because of steadily weakened legs. Finally, at the age of 38 years (1958), he underwent bilateral below-the-knee amputations. Two years later (1960) he received new prosthetic lower legs (which were 22 cm. shorter than his amputated lower legs), thereby reducing his standing height to 213 cm. (Fig. 6). However, stump infections and ulceration persisted. There was no diabetes mellitus and OGTTs were normal. It was also noted that there were sensory deficits at both hands (pin-prick) [22].

**Fig. 7:**
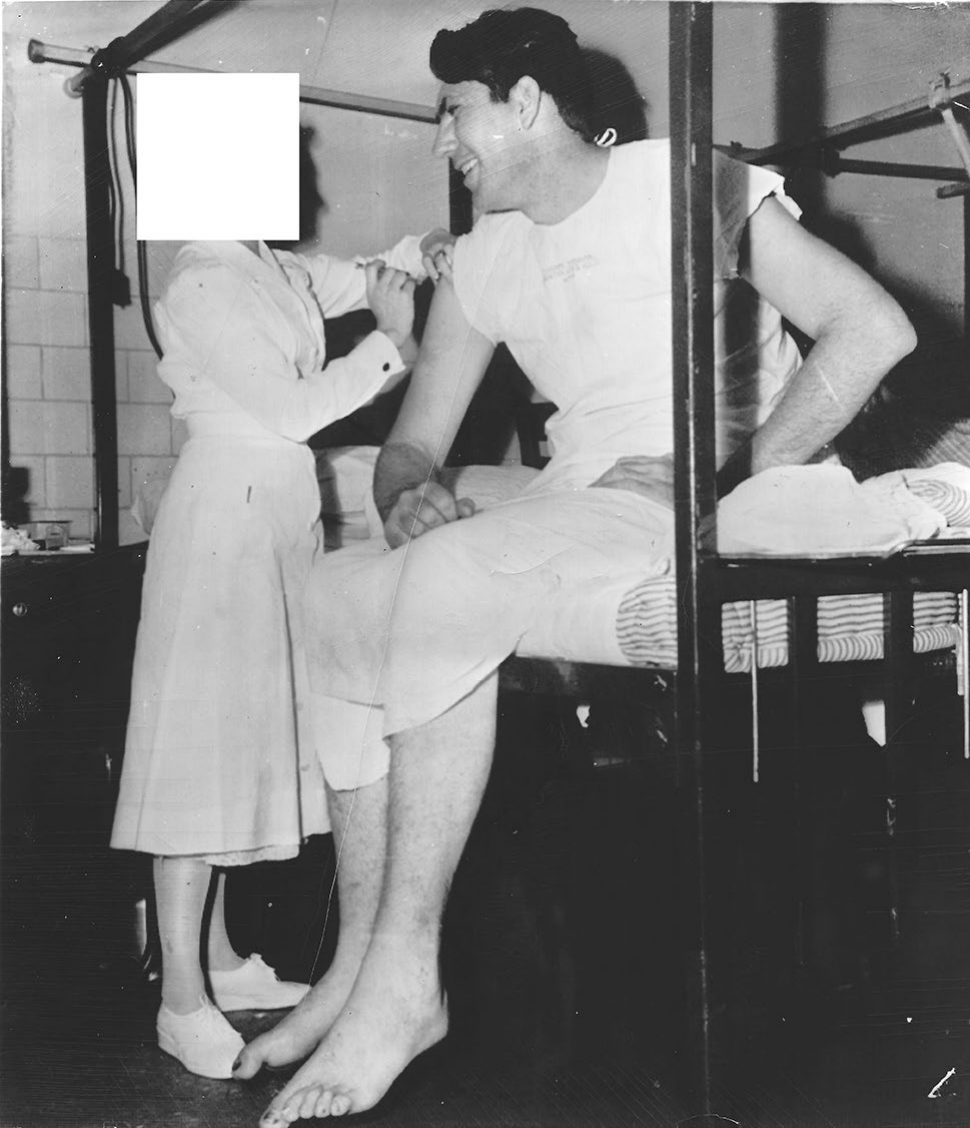
Photograph of the American acromegalic giant Max Edmund Palmer (1927–1984, final height 224.8 cm.) being hospitalized in Charity Hospital in New Orleans, LA, USA, because of an “infected toe”—4 January 1952. Photograph from the private collection of Wouter W. de Herder, which also appeared in the newspaper “The Milwaukee Journal” on 4 January 1952 (among other newspapers) [23]

**Fig. 8:**
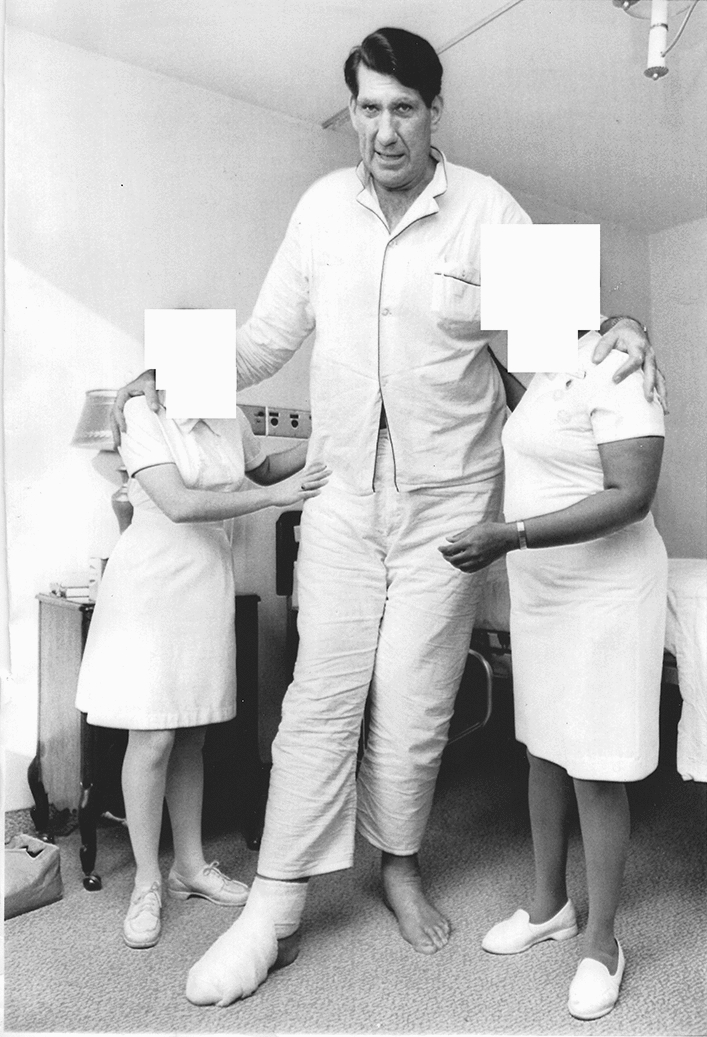
Photograph of the acromegalic giant Max Edmund Palmer (1927–1984, final height 224.8 cm.) being hospitalized in Barnes Hospital in St. Louis, MO, USA, because of a “foot injury” of the right foot—19 February 1970. Photograph from the private collection of Wouter W. de Herder, which also appeared in the newspaper “Tonawanda News” on 19 February 1970 (among other newspapers) [24]

The US acromegalic giant actor, wrestler and evangelist *Max Edmund Palmer* (November 27, 1927–May 17, 1984, the “Clarksdale Giant”, “Paul Bunyan”) was a normal-sized boy until the age of 14 years and then started growing rapidly until the age of 19 years. His final height was 224.8 cm. His maximum weight was 174 kg. According to newspaper articles he was admitted at the Charity Hospital in New Orleans, LA, USA in January 1952 (age 24 years & 1 months) because of an infected toe [23] (Fig. 7) and at the Barnes Hospital in St. Louis in February 1970 (age 42 years & 2 months) because of a foot injury [24] (Fig. 8).

## Neuropathy in acromegaly—acromegalic gigantism

As already discussed by Daughaday and others, paresthesias of the hands caused by the entrapment of the medial nerve in the carpal tunnel (carpal tunnel syndrome) are frequently found in patients with active acromegaly [12, 25–30]. Eventually, also myopathy can be diagnosed [31, 32]. However, as Daughaday also remarked, paresthesias in the lower extremities in combination with sensory deficits, absent reflexes and muscle weakness are very rarely reported in acromegaly, but have been more frequently described in combination with acromegalic gigantism [5, 12, 30–36]. Also, as a consequence, foot ulcers developing in combination with peripheral neuropathy have been described more in combination with acromegalic gigantism than in acromegaly without gigantism [37]. In most cases, diabetes mellitus, or impaired glucose tolerance was absent.

## Neuropathic foot ulcerations—diabetes mellitus

Peripheral neuropathy and neuropathic foot ulcerations are well-known complications of long-standing diabetes mellitus [38–42]. These ulcerations usually affect prominent surfaces of the feet, such as the heel and metatarsal heads, or pressure point areas of high friction in combination with abnormally high pressures on the soles of the feet. Due to the lack of sensation in the area, the patient is much less likely to be able to feel any pain or abnormalities in sensation associated with the ulceration. In diabetes mellitus, it is postulated that reactive oxygen species (ROS) within the tissues can lead to direct nerve injury. In combination with a lack of blood flow this can further lead to Charcot neuroarthropathy [38–42].

## Neuropathic foot ulcerations acromegalic gigantism

The once famous patients with acromegalic gigantism with neuropathic ulcers as described above did not present with diabetes mellitus, or impaired glucose intolerance. Dinn & Dinn collected sural nerve biopsies from acromegalic patients, with and without peripheral neuropathy to study the natural history of peripheral neuropathy in acromegaly [34]. Initially, demyelination in combination with hypertrophic formations affecting the Schwann cell system of the small diameter fibers was found. This could further progress to marked “onion bulb formation” resulting in end-stage neuropathy [34]. Sendur and colleagues measured both static and dynamic plantar variables of 70 patients with acromegaly and analyzed their balance patterns [43]. They found that the mean force on each foot was higher in cases of acromegaly as compared to healthy controls. In the acromegalic individuals, the maximum pressure in the midfoot was higher, while the medial heel maximum pressure was lower as compared to healthy controls. Injury risk was similar. Center of pressure measurements elicited intact balance. In terms of static and dynamic plantar data, there was no difference between patients with active and controlled acromegaly [43].

Jennings and colleagues reported an abnormal high pressure under the metatarsal heads of 2 nondiabetic patients with acromegalic gigantism and peripheral neuropathy [37]. Most patients with acromegalic gigantism will develop deformities of the legs and feet over time, like knee problems and genu valga [44–48], pedes plani [14, 44, 47, 49] and claw feet, or “club-like” feet [13, 35, 50]. Generally, these joint problems seem more severe than those occurring in patients with acromegaly without gigantism [51–56]. In acromegalic gigantism, the combination of leg and foot deformities and a huge weight can result in abnormal high pressure on pressure points of the feet. Most patients with acromegalic gigantism also require custom-made shoes and many times their footwear is of poor quality. Like in patients with diabetic neuropathic ulcers, specific footwear might significantly reduce peak pressures [41].

## Conclusions

Neuropathies of the median and ulnar nerves are well-known complications in patients with active acromegaly. However, also neuropathy of the sural nerve can be found. Particularly in patients with acromegalic gigantism this can lead to hypesthesia and hypalgesia of the lower legs and feet. It is suggested that neuropathic ulcers in the feet in patients with acromegalic gigantism occur more often than in patients with acromegaly but without gigantism. Potential contributing factors in the patients with acromegalic gigantism and neuropathic foot ulcers might be leg and foot deformities, muscle weakness and poor quality footwear. Diabetes mellitus does not seem to play a major role.

## Data Availability

No datasets were used.
